# Ethylene Glycol Poisoning with a Near-Normal Osmolal Gap: A Diagnostic Challenge

**DOI:** 10.7759/cureus.11937

**Published:** 2020-12-06

**Authors:** Moeed Ahmed, Cliff Janikowski, Syed Huda, Aiza Ahmad, Lee Morrow

**Affiliations:** 1 Internal Medicine, Creighton University School of Medicine, Omaha, USA; 2 Pulmonary and Critical Care Medicine, Creighton University School of Medicine, Omaha, USA; 3 Medicine, Upstate University Hospital, Syracuse, USA

**Keywords:** ethylene glycol, osmolal gap, toxicology, alcohol

## Abstract

Ethylene glycol is a colorless, odorless, sweet-tasting liquid commonly found in antifreeze, as well as in industrial agents. It is regarded as one of the toxic alcohols. Ethylene glycol poisoning usually occurs due to ingestion, and its toxicity is mediated by its metabolites, glycolic acid, and oxalate. These metabolites can cause neurological symptoms, gastrointestinal symptoms, and/or renal failure if not diagnosed and treated promptly. The diagnosis can be very challenging as the test used to detect ethylene glycol in the blood may not be readily available or due to an inaccurate history. The treatment of ethylene glycol poisoning consists of supportive care, sodium bicarbonate, and the use of an antidote (ethanol or fomepizole) which inhibits alcohol dehydrogenase and thereby prevents the formation of toxic metabolites. Patients with advanced poisonings may also require dialysis. The diagnosis is usually suggested by a high anion gap metabolic acidosis and an elevated osmolal gap in the setting of a suspected ingestion. Rarely, the osmolal gap may be close to normal which can delay the diagnosis or lead to a misdiagnosis. We report a case of ethylene glycol ingestion with a near-normal osmolal gap.

## Introduction

Ethylene glycol is a colorless, odorless, sweet-tasting liquid commonly found in antifreeze and various industrial agents. Along with methanol, it is regarded as one of the toxic alcohols. Ethylene glycol poisoning mostly occurs due to ingestion, either accidental or intentional. According to the Annual Report of the American Association of Poison Control Centers' National Poison Data System, there were 6,374 reported cases of ethylene glycol toxicity in 2016 [1]. Most of these cases were in adults over the age of 20 years and were intentional. Ethylene glycol toxicity is mediated by its metabolites, primarily glycolic acid and oxalate [2]. To prevent the clinical complications of ethylene glycol intoxication, early identification and intervention are important to reduce the production of these toxic metabolites [3]. The treatment of ethylene glycol poisoning consists of supportive care, sodium bicarbonate, and consideration of an antidote, such as ethanol or fomepizole. These agents inhibit alcohol dehydrogenase, thereby preventing the formation of toxic metabolites. In extreme cases (pH < 7.25, acute kidney injury (AKI), serum ethylene glycol level > 50 mg/dL), the accumulation of toxic metabolites is highly likely and antidotes alone are insufficient. In these cases, patients also require dialysis to directly remove ethylene glycol and toxic metabolites. Ethylene glycol poisoning is classically associated with a high anion gap metabolic acidosis (HAGMA). In poisoned patients, neurological and gastrointestinal symptoms predominate early on, while renal failure and death occur if not diagnosed and treated promptly. The diagnosis is usually suggested by HAGMA and an elevated osmolal gap (with or without AKI) in the setting of a suspected ingestion. Rarely, the osmolal gap may be close to normal which can delay the diagnosis or lead to a misdiagnosis. We report a case of ethylene glycol ingestion with a near-normal osmolal gap. 

## Case presentation

An 85-year-old man with a past medical history of dementia presented to the emergency department with altered mental status, restlessness, and an elevated creatinine of 1.4 mg/dL (baseline 1.2 mg/dl). History was difficult to obtain and his baseline mental status was not known. Vital signs were normal, and the physical examination was remarkable only for his cognitive deficits.

Computed tomography (CT) scan of the head did not reveal any acute abnormality. Laboratory workup revealed a white blood cell (WBC) count of 16.9 K/uL, anion gap 21 mEq/L, arterial blood pH 7.26, serum bicarbonate 9.3 mmol/L, and lactic acid 2.2 mmol/L. The serum osmolal gap was slightly elevated at 12 mOsm/kg (normal: < 10 mOsm/kg). Laboratory studies within normal limits included: urinalysis, urine drug screen, blood ethanol, beta-hydroxybutyrate, acetaminophen, salicylate, thyroid-stimulating hormone (TSH), vitamin B12, folate, thiamine, urine osmolality, sodium, potassium, chloride, and blood urea nitrogen. Blood cultures were drawn, given the leukocytosis and suspicion of infection. Blood ethylene glycol and methanol levels (sent out tests) were ordered, and he was admitted to the intensive care unit (ICU). 

The patient was treated with intravenous (IV) fluids and empiric antibiotics. Given a high clinical suspicion for toxic alcohol ingestion, fomepizole was initiated. Nephrology was consulted. Per their recommendation, the fomepizole was discontinued after two days, concurrent with improvement in his mental status and resolution of the anion and osmolal gaps. Subsequent additional history from his family increased the likelihood of toxic alcohol ingestion. His ethylene glycol level eventually resulted in a level of 3.1 mg/dL. The blood methanol level was unremarkable. 

## Discussion

The diagnosis of ethylene glycol poisoning can be very challenging. Solid clinical judgment and a high index of suspicion are critically important as therapy must be started early and ethylene glycol level is usually a send out test that takes days to be reported. Early diagnosis of ethylene glycol poisoning is hindered by the inability to obtain an accurate history as patients often present with altered mental status or may avoid reporting alcohol ingestion. As such, a presumptive diagnosis requires recognition of correlating signs and symptoms and particular attention to the available labs.

Ethylene glycol poisoning usually presents with HAGMA and an elevated osmolal gap. Other conditions that can be associated with HAGMA include ketoacidosis, lactic acidosis, ethanol intoxication, acetaminophen or salicylate toxicity, uremia, and renal failure. Other than toxic alcohol ingestion, ketoacidosis, lactic acidosis, and chronic kidney disease can also cause a high osmolal gap. The osmolal gap is calculated as measured serum osmolality/calculated serum osmolality. The serum osmolality is calculated using the concentrations of major plasma solutes which include sodium (measured in mmol/L), glucose (measured in mg/dL), and urea (measured in mg/dL). The formula commonly used to calculate serum osmolality (Sosm) is: Sosm = (2 x serum sodium) + (glucose/18) + (blood urea nitrogen/2.8) [4]. If ethanol is present in blood, then the formula becomes: Sosm = (2 x serum sodium) + (glucose/18) + (blood urea nitrogen/2.8) + (ethanol/3.7).

An elevated serum osmolal gap exists if the measured osmolality exceeds the calculated osmolality by more than 10 mOsm/kg. An elevated osmolal gap indicates the presence of solutes in the serum (other than sodium salts, urea, and glucose), such as toxic alcohols [5]. As the test to directly measure the ethylene glycol level is usually not readily available, the osmolal gap serves as a rapid surrogate test. In the appropriate clinical setting, detection of osmotically active substances in the plasma of an at-risk patient is sufficient to justify the cost of starting appropriate interventions [6]. The main limitation of the serum osmolal gap is that it is insensitive in late presentations, as most of the parent alcohol has already been metabolized [7]. In our case, the patient presented a diagnostic challenge, given his inability to provide any substantial history, a nonspecific HAGMA, and a near-normal serum osmolal gap of 12 mOsm/kg, a value that was not particularly convincing for or against the diagnosis of toxic alcohol ingestion. In retrospect, our patient’s relatively normal osmolal gap likely reflected that he had a modest ingestion, given his eventual serum ethylene glycol level and relatively benign clinical course.

Metabolism of ingested methanol or ethylene glycol causes a progressive decline in the osmolal gap. When the parent molecule is metabolized to acid metabolites, such as formic acid (in the case of methanol) or glyoxylic and oxalic acid (in the case of ethylene glycol), their osmotic contribution disappears because each molecule of organic acid produced generates an equimolar disappearance of bicarbonate. As a result, the increase in organic anion osmoles are matched by a similar fall in bicarbonate osmoles. Therefore, the serum osmolal gap estimates the molar quantity of the uncharged parent alcohol molecules but not their acidic metabolites. The result of these metabolic effects is that the osmolal gap reflects the concentration of the ingested alcohol and falls with its metabolism, while the high anion gap acidosis is the result of metabolism and increases as the alcohol is converted to an acidic product. Only the osmolal gap may be present in patients who present very early after ingestion, and only the high anion gap metabolic acidosis may exist in patients who present very late after ingestion.

The following criteria are used to start antidote therapy for ethylene glycol poisoning [8]: serum ethylene glycol > 20 mg/dL (3.2 mmol/L, documented recent ingestion of toxic amounts of ethylene glycol and an osmolal gap > 10 mOsm/L, suspected ethylene glycol ingestion, and at least two additional criteria, including arterial pH < 7.3, serum bicarbonate < 20 mmol/L (mEq/L), osmolal gap > 10 mOsm/L, and/or oxalate crystalluria.

In our case, the ethylene glycol level was mildly elevated at 3.1 mg/dL, indicating that either there was a substantial ingestion and the ethylene glycol had substantially metabolized already or it was a milder ingestion. As the result was not available in real-time, we opted to start fomepizole based on high clinical suspicion and neurologic manifestations (altered mental status). This decision was also further supported by the substantial metabolic acidosis (serum bicarbonate of 9.3 mmol/L and arterial blood pH of 7.26). It should be noted that oxalate crystals in the urine, a late manifestation of ethylene glycol poisoning, were absent in our case. The patient’s rapid improvement in mental status and avoidance of hemodialysis also justified our early use of fomepizole.

## Conclusions

The workup for HAGMA should include an evaluation of the serum osmolal gap, particularly in the setting of suspected toxic alcohol ingestion. Although uncommon, toxic alcohol ingestion can present without an osmolal gap, as this case illustrates. Accordingly, the absence of an elevated osmolal gap should not discourage treatment for toxic alcohol ingestion when the clinical suspicion is sufficiently high.
